# Multiple Dental Agenesis with an Impacted Maxillary Canine in an Early Medieval Dog (*Canis lupus familiaris*) from Wolin, Poland—A Case Study

**DOI:** 10.3390/ani16081219

**Published:** 2026-04-16

**Authors:** Piotr Baranowski, Katarzyna Grocholewicz, Aleksandra Gawlikowska-Sroka

**Affiliations:** 1 Department of Animal Anatomy and Zoology, Faculty of Biotechnology and Animal Sciences, West Pomeranian University of Technology in Szczecin, Klemensa Janickiego 33, 71-270 Szczecin, Poland; 2 Department of Interdisciplinary Dentistry, Pomeranian Medical University, al. Powstańców Wlkp. 72, 70-111 Szczecin, Poland; katarzyna.grocholewicz@pum.edu.pl; 3 Department of Anatomy, Pomeranian Medical University, al. Powstańców Wlkp. 72, 70-111 Szczecin, Poland; aleksandra.gawlikowska.sroka@pum.edu.pl

**Keywords:** archaeozoology, dog, impacted canine, dental agenesis, palaeopathology, radiography, Wolin

## Abstract

Dental developmental anomalies are well documented in clinical veterinary medicine but remain rarely reported in archeological dogs. This study presents a radiologically confirmed case of an unerupted left maxillary canine associated with the absence of an alveolus for the left maxillary first molar and incisors in a dog skull from early medieval Wolin. This study aimed to determine whether the observed absence of teeth resulted from congenital agenesis, developmental arrest, ante-mortem loss, or post-depositional processes. Radiographic examination revealed a fully formed but unerupted canine, while the M^1^ region exhibited a smooth bony surface without reactive remodeling, periapical radiolucencies, or signs of ante-mortem tooth loss. Differential diagnosis did not support canine agenesis, ante-mortem loss, or taphonomic damage as primary explanations. The findings most strongly support a congenital or very early developmental origin of the observed alterations. The estimated age of the individual (7–10 years) and the absence of secondary pathological changes suggest that these anomalies did not significantly impair masticatory function. Owing to the single-case nature of the material, broader population-level inferences cannot be made. This case underscores the methodological importance of radiographic imaging in archeological dental research and suggests that alveolar absence should not be automatically equated with impaired survival or poor health in this individual.

## Simple Summary

This study documents a rare co-occurrence of an unerupted left maxillary canine (C^1^ sin.) and the absence of an alveolus for the left maxillary first molar (M^1^ sin.) and incisors in an early medieval dog from Wolin (ca. 1100–1150 AD). The aim was to establish the most plausible etiological explanation for these findings based on integrated macroscopic, osteometric, and radiographic analyses. No evidence of inflammatory, traumatic, or taphonomic alteration was observed, allowing exclusion of ante-mortem or post-mortem tooth loss. Differential diagnostic criteria support a congenital or very early developmental origin of the observed changes. Radiographic analysis supported the differential diagnosis of the observed dental anomalies and helped clarify the origin of the alveolar absence. Due to its single-case nature, no broader population-level conclusions can be drawn about dental anomalies in archeological dogs.

## 1. Introduction

### 1.1. Clinical and Veterinary Context of Dental Anomalies in Dogs

Dental anomalies in domestic dogs are an important aspect of veterinary research, as they can significantly affect masticatory function and the overall health of animals. Contemporary clinical literature provides detailed descriptions of congenital, developmental, and post-traumatic dental disorders in canines [1,2,3,4,5,6,7]. These studies are dominated by radiological and clinical analyses of cases involving impacted teeth, agenesis, supernumerary teeth, and eruption sequence disorders, which allow precise determination of the etiology of the changes, their functional consequences, and therapeutic options.

Anomalies such as missing [8,9] or impacted teeth [1,10], supernumerary teeth [11,12,13], and eruption sequence disorders [2,14] may provide information on health, selective pressure [15], and human–animal relationships [16,17,18]. Data from wild species indicate that some of the mechanisms leading to tooth loss are universal [19,20], which justifies the cautious use of interspecies analogies in the interpretation of archeological materials.

### 1.2. Comparative Evidence from Wild Canids

Analysis of dental anomalies in wild canids provides an important comparative context for interpreting changes observed in the domestic dog. In contrast to clinical data, non-clinical material, encompassing free-living populations and museum collections, allows the capture of the natural spectrum of dental variation resulting from the interaction of genetic, environmental, and demographic factors. Of particular importance here are studies on European members of the *Canidae* family, such as the wolf (*Canis lupus*), red fox (*Vulpes vulpes*), and raccoon dog (*Nyctereutes procyonoides*).

The most comprehensive quantitative data pertain to the red fox, which is one of the best-studied species with respect to dental variation. An analysis of a large population sample of 1453 specimens from Poland revealed the presence of anomalies in approximately 13.5% of individuals, with oligodontia (7.2%) being the dominant category, along with changes in crown morphology, enamel hypoplasia, and the presence of additional roots [21]. Similar results were obtained in a Czech population, where deviations from the norm were found in 21.7% of individuals; however, a significant portion of these fell within the range of genetically based morphological variants rather than clear-cut pathologies [22]. This highlights the need to distinguish between “anomalies” and “variability” in the analysis of osteological material. Scandinavian studies further emphasize the importance of environmental factors. The high incidence of dental trauma, wear, and crown fractures is directly linked to lifestyle and foraging strategy [20].

In the case of wolves, the data are more diverse, encompassing both pathological and population-genetic aspects. An analysis of the Scandinavian population revealed a significant increase in the frequency of anomalies over several decades (from 13% to 40%), which is interpreted as resulting from inbreeding and a decline in the population’s genetic health [23]. These results underscore the importance of demographic factors in shaping the dental phenotype. In turn, studies of the Iberian wolf, based on museum specimens, indicate a high prevalence of acquired pathologies, such as periodontitis, tooth wear, and fractures, which are closely linked to age, diet, and foraging behavior [24]. The presence of agenesis and tooth loss of various etiologies demonstrates that even in wild populations, the boundary between developmental and acquired changes can be fluid.

Data on the raccoon dog (*Nyctereutes procyonoides*), in which a relatively high incidence of dental anomalies has been reported, provide important additional context. Approximately 25% of the analyzed skulls exhibited anomalies, with hypodontia (approx. 20%) predominating over hyperdontia [25]. The characteristic location of the missing teeth (premolars and third molars) suggests the existence of stable patterns of dental reduction that may have evolutionary and functional significance. In a broader comparative context, these observations correspond with clinical data on hypodontia in humans, where the most commonly missing teeth are those that develop latest in a given morphological series, which is interpreted as an effect of developmental constraints and the modulation of odontogenesis processes [26].

Against the backdrop of the above observations, studies on hybridization within canids are particularly interesting. Morphological analyses of hybrids indicate an increased frequency of dental anomalies, such as crowding or malalignment of teeth, which is interpreted as the result of developmental disorders arising from the mixing of distinct gene pools [27]. These phenomena are directly relevant to research on the early stages of dog domestication and fit into a broader model of phenotypic instability observed in mammalian hybrids [28]. Consequently, dental anomalies observed in the archeological record cannot be unambiguously interpreted as an indicator of domestication, but should also be considered in the context of hybridization and gene flow between wild and domesticated populations [29,30,31].

### 1.3. Developmental Basis of Dental Anomalies

Contemporary research on the etiology of hypodontia clearly indicates its multifactorial nature. Dental development is controlled by a complex network of genetic and environmental interactions, including, among others, homeobox genes (particularly MSX1 and MSX2) and signaling cascades regulating epithelial–mesenchymal interactions during odontogenesis [26]. Disruptions in these processes can lead to a wide spectrum of phenotypes, ranging from isolated tooth absence to severe forms of oligodontia, often associated with systemic syndromes. At the same time, the variable expression of these traits, observed even in individuals with identical genotypes—as confirmed by studies of monozygotic twins (cited by Larmour et al. [26])—indicates the significant role of environmental and epigenetic factors.

Additionally, numerous studies highlight the co-occurrence of hypodontia with other dental anomalies, such as microdontia, tooth retention, or eruption disorders. These associations may result from shared developmental and genetic mechanisms, including, among others, signaling disorders within the dental lamina [26]. Such correlations are also interpretive in archaeozoological material, where the co-occurrence of various anomalies may indicate systemic developmental disorders rather than isolated cases of pathology.

### 1.4. Broader Mammalian Perspective on Odontogenesis Variability

A compilation of data for various canid species indicates that dental anomalies are a common phenomenon, although their frequency and nature vary greatly. In many cases, the observed changes fall within the range of natural population variation and do not necessarily have a pathological nature in the strict sense. At the same time, factors such as inbreeding, environmental pressure, diet, or hybridization can significantly increase the frequency and severity of abnormalities.

In a broader comparative context, these data fit into the general pattern of odontogenesis variability in mammals. Similar trends have been observed, for example, in horses [32], bats [19], and pigs [33], where the recurrence of specific types of anomalies is interpreted as a manifestation of microevolutionary processes or strong genetic control of dental development. Consequently, dental anomalies in dogs should be analyzed not only in the context of clinical pathology but also as part of a broader, interspecies continuum of morphological variation. Studies on bats further indicate a strong phylogenetic component in tooth reduction and retention [19], and in wild boars and domestic pigs, recurrent agenesis of the first premolar has been documented, interpreted as a possible microevolutionary trend [33]. Reports of hypodontia and hyperdontia in beavers [34] confirm that the spectrum of anomalies observed in dogs falls within a broader, mammal-wide pattern of odontogenesis variability.

### 1.5. Anthropological Background

Tooth development and eruption in mammals exhibit a high degree of homology [35]; therefore, anthropological data can provide a useful comparative framework. In human populations, retained maxillary canines and the absence of fully formed alveoli are among the best-documented anomalies [36,37,38] and are often asymptomatic, becoming apparent only on radiographs [39]. Analogous phenomena are observed in veterinary dentistry, where impacted teeth in dogs may remain clinically undetected for a long time, particularly in brachycephalic breeds [40,41]. The purpose of integrating human and animal data is not to compare prevalence rates, but to better understand the biological mechanisms leading to the absence of a tooth or tooth socket in bone material.

### 1.6. Archaeozoological Context

In contrast to extensive clinical documentation, analyses of dental anomalies in archeological materials remain relatively rare [9,17,42,43,44]. This is primarily due to the fragmentary preservation of cranial material and the historical focus of archaeozoological research on features relevant to dating and taxonomic identification. The proportion of bones with pathological changes in faunal assemblages is usually small—for Roman sites in the Mediterranean region, it did not exceed 3% [45], and in many other contexts, it was even lower [46]. Craniofacial malformations and dental abnormalities typically account for about 1% of the material [47,48,49,50,51], which contrasts with the more frequently recorded chronic post-traumatic or degenerative changes [52,53]. Despite their low frequency, these findings provide important information about animal diseases, living conditions, husbandry practices [54], and human–animal relationships in the past [46,55,56,57].

### 1.7. Archaeozoological Evidence from Wolin

The early medieval settlement at Wolin [58,59,60], located on the southern coast of the Baltic Sea [61,62], yielded a substantial collection of animal remains [63,64,65], including well-preserved specimens with pathological or post-traumatic changes in the axial skeleton, as well as in the stylopodium, zeugopodium, and autopodium of cattle, pigs, horses, sheep, and dogs. Also represented are specimens with post-traumatic changes in the vertebrae, ribs, tibia, and fibula [51,66], as well as evidence of fractures of the zygomatic bone in horse skulls or lesions resulting from a chronic disease process typical of actinomycosis (*Periostitis actinomycotica*) on the mandible of a pig. Injuries resulting from impact are also present on the skulls of the aforementioned animal species and are likely non-fatal in dogs [67]. While pathological changes in the dentition were noted in cattle and pigs from Wolin, none were recorded in dogs [51]. Meanwhile, among this material, a specimen exhibiting a combination of missing teeth and a retained canine is particularly noteworthy. Such a co-occurrence of developmental abnormalities has not been documented in archeological canid remains, making this case an important contribution to research on canine health in early medieval Europe.

### 1.8. Functional and Cultural Implications

Given the growing role of dogs in the Middle Ages as guards, companions, and working animals, analyses of dental anomalies can provide broader contextual information regarding selective breeding, population structure, and living conditions [54,68,69,70,71,72]. Situating this specimen within the broader body of archaeozoological and veterinary literature will deepen our understanding of the biological and cultural factors influencing canine dentition over time.

### 1.9. Research Gap

In archaeozoology, there are studies on dental trauma, tooth loss, and general dental changes in dogs; however, detailed descriptions of the coexistence of an impacted canine and missing M1, I1–I3 teeth in archeological material that have been confirmed by radiography are very rare or have not been published in a comprehensive form. This makes the present case a unique clinical and paleopathological record of a dog from the early Middle Ages.

In the authors’ opinion, the use of radiology/imaging to distinguish between agenesis and early developmental loss in archaeozoology remains limited. New pilot studies highlight the value of a standardized clinical protocol for recording the condition of teeth in archeological animals and indicate that imaging (X-ray, CT) significantly increases diagnostic certainty; these standards are becoming increasingly common.

Due to the lack of metric and population-based comparisons for medieval dog populations from the Baltic region in the analysis of dental anomalies, the present study complements the results of earlier research on material from Wolin [67], focusing specifically on the correlation of odontogenetic anomalies with the morphological type and age of the dog whose skull was found. The obtained osteometric data and contextual information (the emporium of Wolin) may complement earlier findings.

Currently, there is a need for an interdisciplinary approach—clinical, archaeozoological, paleopathological, and, if possible, genetic—in interpreting the causes of anomalies. Although the mechanisms of retention and agenesis are well described in clinical and veterinary literature, they are rarely directly linked to archeological and population data. The present study proposes a model that integrates these perspectives.

### 1.10. Research Objectives

The main objective of this study was to determine the most likely etiological explanation for the coexistence of an unerupted maxillary canine (C1 sin.) and the absence of the M1 sin., I1 sin., I1–I3 dex. in a medieval dog from Wolin, based on integrated macroscopic, radiographic, and osteometric analyses. In particular, the study aimed to determine whether the observed pattern is consistent with congenital agenesis, early developmental arrest, tooth bud arrest, early loss, or post-mortem processes, using differential diagnostic criteria drawn from the clinical and paleopathological literature.

## 2. Materials and Methods

### 2.1. Study Material and Specimen Selection

**Figure 1:**
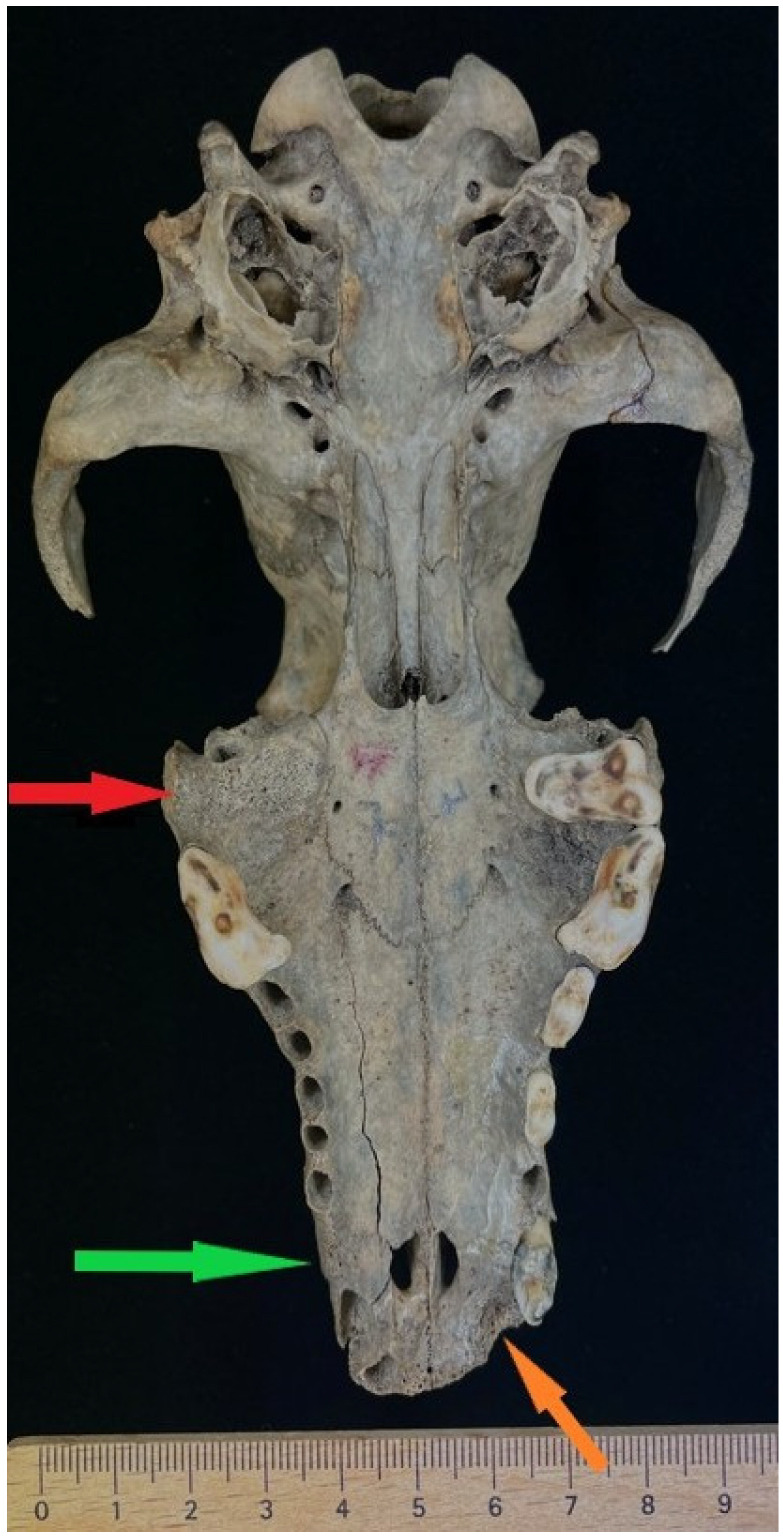
Ventral view of the skull of a dog. The red arrow indicates the absence of the alveolus of the first molar tooth (M1). The green arrow marks the absence of the canine alveolus. The orange arrow shows a depression corresponding to the alveolus of the third incisor, with a distinctly roughened internal surface.

Among the 13 dog skulls found at sites in Wolin Town and the surrounding area—Wolin Silver Hill—one specimen with inventory number 2493 AR955/7 was selected. It is housed in the archaeozoological collection of the Department of Animal Anatomy and Zoology (access available upon reasonable request) and dates from the period between 1100 and 1150 CE [67], characterized by the absence of tooth sockets C1, M1, I1 (left), and I1–3 (right) (Figure 1).

Additionally, asymmetry in the morphology of the left and right palatine fissures is evident. A longitudinal fracture is present along the alveolar margin of the left maxilla, extending for approximately 47.63 mm.

Due to the uniqueness of this case compared to the other 12 specimens, in which no dental anomalies were found, the skull was subjected to radiographic examination. Analysis of the radiograph revealed the presence of a well-formed canine retained in the left maxilla, with a confirmed absence of tooth M1.

### 2.2. Macroscopic Examination, Age Estimation, and Sex Determination

The skull underwent detailed visual examination and osteometric measurements. Given that occlusal wear patterns may be significantly influenced by diet and living conditions, age estimation was therefore additionally based on the evaluation of cranial suture closure as an independent osteological indicator. For this purpose, the location of synchondroses and suturae was utilized [67,73,74]. It was determined that the skull belonged to an individual whose age at death ranged between seven and ten years. It was assumed that the age range may result from allometric and ecological influences correlated with the timing and pattern of cranial suture closure [75,76]. Due to the absence of the postcranial skeleton, including the baculum and long bones [77,78], the most appropriate available approach for sex determination in this individual was that proposed by Trouth et al. [79].

### 2.3. Measurement Procedure

Both whole bones and measurable fragments were measured on the skull. Basic measurements were taken at the points outlined in the guide “A guide to the measurement of animal bones from archeological sites” [80] three times using a digital caliper and a hinged bow compass (craniometer) with an accuracy of 0.01 cm, and the average was calculated. For features not included in von den Driesch’s guide [80], the procedure described in a previous study [67] was followed. The measurement error ranged from 1% to 3%. Calculations to determine the sex of the skull donor were performed based on the ratio of the distance between the two temporoparietal fissures at their most lateral points to the distance between the base and a line drawn between the two most medial points of the foramen magnum in the midline [79]. The index was 113.62. Given that index values below 123 indicate a male skull and values above 136 indicate a female skull, the obtained index classifies skull 2493 AR955/7 as male.

### 2.4. Radiographic Imaging and Protocol

X-ray Imaging. Sample and Imaging Material.

**Figure 2:**
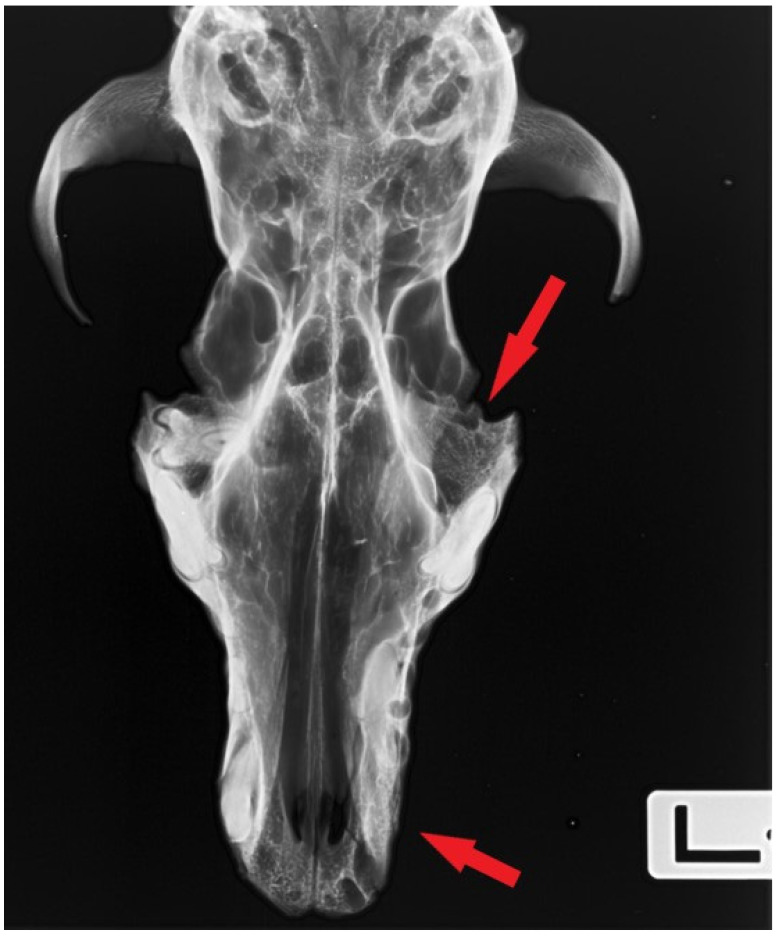
Dorsoventral (DV) projection of the dog’s skull. Arrows indicate the absent M1 tooth socket and the unerupted (impacted) maxillary canine.

**Figure 3:**
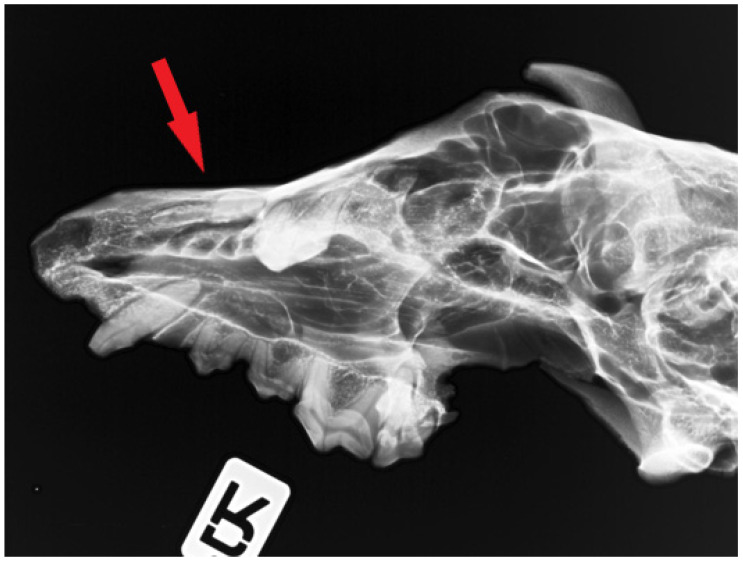
Radiographic image of the skull of the same dog obtained in a right ventral–left dorsocaudal oblique projection (20°). The arrow indicates the region of interest in the rostral part of the maxilla.

**Figure 4:**
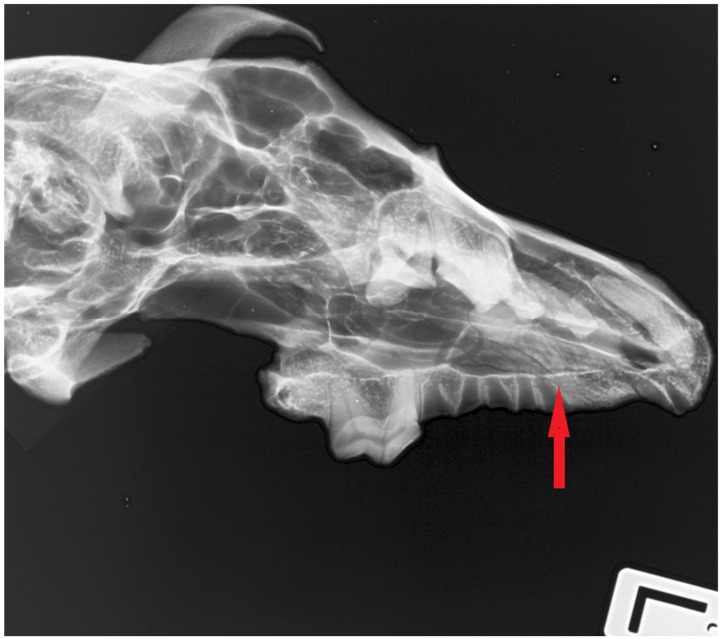
Radiographic image of the skull of the same dog obtained in a left ventral-right dorsocaudal oblique projection (20°). The arrow indicates the region of interest in the rostral part of the maxilla.

Image documentation of a dog skull dating to 1100–1150 AD included a macroscopic image of the skull base (Figure 1), X-rays of the dorsal view (Figure 2), and X-rays of the right and left sides of the skull. The right side was imaged in a dorsocaudal-ventral oblique projection (20°), and the left side in a dorsocaudal-ventral oblique projection (20°) (Figure 3 and Figure 4). Radiographic examination was performed to assess the internal bony structure, the morphology of the alveolar process, and to identify any remodeling changes.

### 2.5. Radiographic Parameters and Setup

The study was performed using a Control-X Medical system (bone–lung system) with the following parameters: anode voltage of 130 kV with automatic exposure control (AEC), a total filter thickness of 1.5 mm Al (1.2 mm Al + 0.3 mm Al), and internal filtration in the range of 0.5–1.0 mm Al. The source-to-detector distance (SID) is typically set at 100–120 cm, typically used in live animals, and images were digitally recorded in DICOM format, allowing for subsequent contrast analysis and assessment of subtle structural details.

Unlike clinical imaging of live animals, the study material did not contain soft tissue, which in vivo constitutes a significant absorption barrier for X-rays. The absence of muscle, adipose tissue, and skin resulted in increased radiation transmission and improved visualization of the trabecular bone structure. With this in mind, radiographs were taken using right and left lateral oblique sections of the skull to evaluate tooth C1 and the M1, I1–3 regions. Exposure parameters ranged from 55 to 75 kV and 1–4 mAs, with a source-to-image distance (SID) of 100–110 cm and an internal filtration of 0.5–1.0 mm Al.

### 2.6. Evaluation and Interpretation

Due to the lack of soft tissue attenuation, lower exposure parameters than those typically used in clinical veterinary radiography were sufficient to achieve adequate penetration and clear delineation of cortical and trabecular structures. Although dry archeological bone may exhibit increased radiographic opacity due to demineralization, digital DICOM acquisition allowed for tonal compensation and detailed structural analysis. The selected parameters provided sufficient contrast resolution to distinguish hypoplasia from post-mortem loss without introducing motion artifacts or exposure-related distortions.

## 3. Results

### 3.1. Morphological Description

The skull of the dog with inventory number 2493 AR955/7 is preserved in a condition that allows for a complete morphological and osteometric analysis (Table 1 and Table 2). Its total length (*Acrocranium-Prosthion*) is 182.0 mm, the condylobasal length is 171.0 mm, and the skull base length (*Basion-Prosthion*) is 162.0 mm, which places the individual in the group of medium to large dogs with mesocephalic proportions. The estimated Wyrost–Kucharczyk coefficient [81], based on the *Basion–Etmoideum* length, indicates that this individual was 51.81 cm tall (Table 1).

The length of the visceral skull (*Nasion–Prosthion* = 89.88 mm) and the length of the muzzle (oral border of the orbits (median)—*Prosthion* = 81.95 mm) are proportionally consistent with values observed in medieval working dogs from the southern Baltic coast (Table 2).

The zygomatic width (*Zygion–Zygion* = 105.86 mm) and the width of the braincase (*Euryon–Euryon* = 59.12 mm) indicate a moderately massive head structure. The cranial indices (*Zygion–Zygion* × 100/*Acrocranion–Prosthion* = 58.16 and *Euryon–Euryon* × 100/*Acrocranion–Nasion* = 64.18) confirm a mesocephalic morphological type, without features of extreme brachycephaly or dolichocephaly (Table 3).

### 3.2. Skull Preservation and Taphonomic Condition

**Figure 5:**
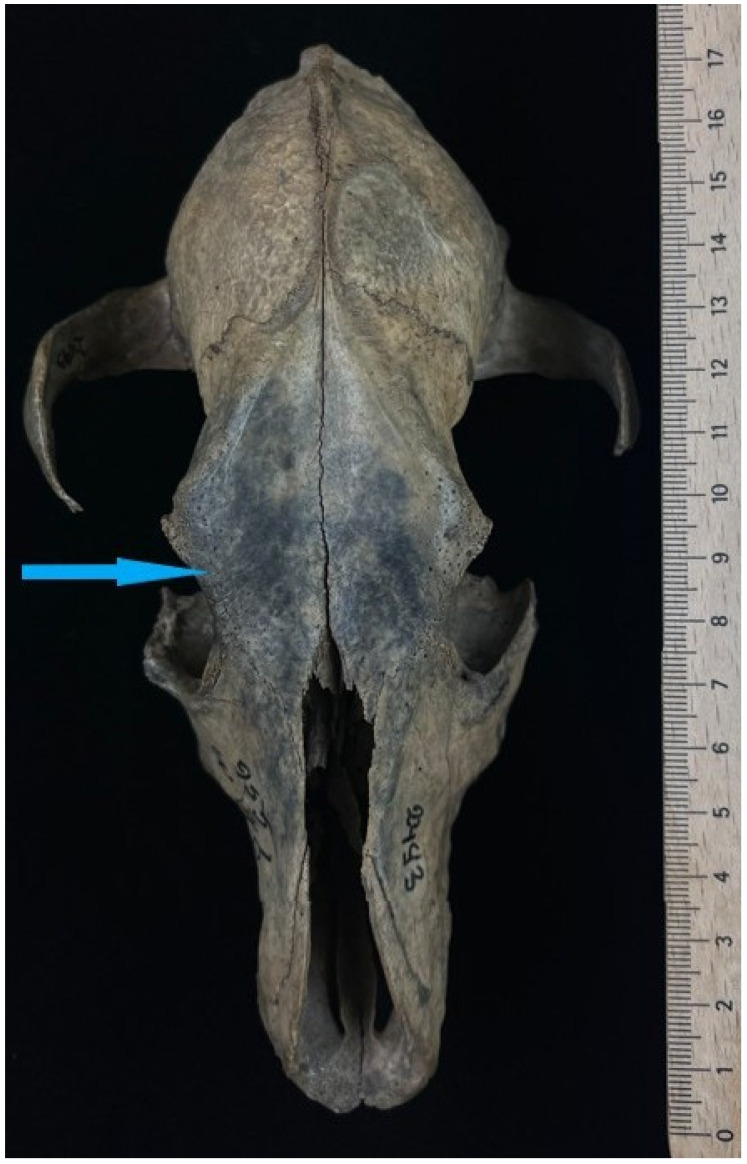
Dorsal view of a dog’s skull. Taphonomic features visible include discoloration of the frontal bone.

The skull is preserved in a moderate state of preservation, with clear evidence of post-depositional damage. This is evidenced by the absence of extensive fragmentation and complete bone disintegration of the bone structure, as well as the integrity of the present bone complexes. The major parts of the cranial and facial skeleton are preserved, although the specimen exhibits fragmentation and bone loss, particularly in the facial region and the palate. The bone surface shows signs of surface weathering manifested as fine cracks, flaking, and localized porosity and brittleness. Overall, it is a fully anatomically identifiable specimen, retaining morphological features and clear bone surfaces (Figure 5).

### 3.3. Cranial and Visceral Components

**Figure 6:**
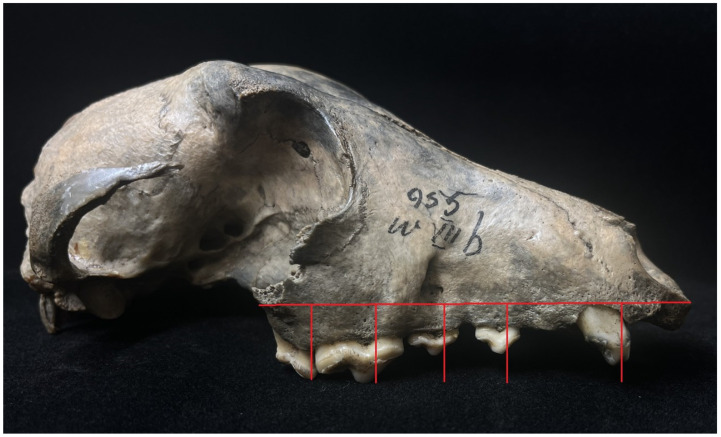
Canis cranium right side view. The horizontal red line represents the occlusal plane (base of the alveolar process), serving as a reference for evaluating the inclination of the teeth. The vertical red lines indicate the long axes of individual teeth in the right maxillary dental arch, from the molar (M1) to the canine (C). The molar and carnassial teeth are positioned nearly perpendicular to the occlusal plane, whereas the canine shows a characteristic anterior inclination, consistent with the masticatory biomechanics of carnivores. The vertical lines intersect the centers of the tooth crowns and correspond with the axes of the alveoli, indicating anatomically correct positioning. No evidence of pathological displacement, rotation, or malalignment is observed; the teeth are stably seated within their sockets.

The cerebral part is preserved in a complete state, with minor superficial erosions present. On the ventral part of the cerebral skull, defects in the walls of the left and right tympanic cavities are visible (Figure 1). In contrast, the visceral part of the skull is incomplete. The nasal bones, as well as the bodies and arches of the zygomatic bones, are missing (Figure 5). The body of the left zygomatic bone is completely absent. A fragment of the body of the right zygomatic bone is preserved in its oral segment, measuring 23.68 mm × 12.64 mm. This fragment is fused with the maxilla over a length of 21.36 mm (Figure 6). The absence of the nasal bones and zygomatic elements and the defects in the thin and delicate walls of the tympanic cavity indicate that the cause of the damage was most likely pressure from the ground.

### 3.4. Post-Depositional Damage and Fracture Patterns

A fracture line on the facial surface of the left maxilla indicates bone fragility is indicated by a fracture line on the facial surface of the left maxilla, extending from the oral margin of the orbit obliquely toward the undeveloped canine alveolus, with a length of 56.70 mm. On the ventral side of the visceral part of the skull, along the alveoli starting from the oral margin of the maxilla to the boundary marked by the palatomaxillary suture (*sutura palatomaxillare*), another fracture line runs for a length of 47.63 mm (Figure 1). Both fractures are most consistent with post-depositional mechanical damage, in contrast to the distinct separation along the frontal suture (*sutura interfrontalis*). This separation is most likely related to diagenetic processes, including bone dehydration after excavation, associated with stress and microcracking along natural lines of fusion.

### 3.5. Taphonomic Interpretation

In general, the cranial vault is better preserved than the facial skeleton, suggesting differential taphonomic exposure. This may reflect partial burial or varying microenvironmental conditions (Figure 5). No clear signs of rodent gnawing or plant root activity were observed, which supports stable burial conditions in a moist, anaerobic substrate characteristic of early medieval Wolin. The dark discoloration on the teeth appears to result from mineralization of soil sediments, not dental caries in the strict sense.

### 3.6. Dental Preservation and Wear Patterns

The maxillary dentition is incomplete. Some teeth were lost post-mortem, as evidenced by empty alveoli and mechanically damaged alveolar margins. The teeth present in the right dental arch are the canines, P2, P3, P4, and M1. The left dental arch contains only the P4 tooth. The preserved teeth exhibit morphology consistent with typical canine dentition. The premolars have elongated, blunted crowns, and their chewing surfaces—as well as those of the molars—are partially worn down. No distinct deformations of the crown shapes are observed; wear is evident and includes flattening of the crown cusps, localized dentin exposure, and reduction in the sharp edges typical of predator teeth. This indicates moderate to advanced dental wear, which may be associated with a diet containing abrasive components. The degree of wear on the molar crowns (P4, M1), assessed using the Grant scale [82], indicates advanced exposure of secondary dentin. This, in turn, correlates with the studies by Horard-Herbin [83] on dog populations in early urban centers and may indicate a highly abrasive diet typical of synanthropic individuals.

### 3.7. Alveolar Morphology and Asymmetry

Macroscopic analysis revealed the absence of the alveolar socket of the first maxillary molar on the left side (M1 sin.). A normally developed M1 alveolar socket was present on the right side (length of the M1 (dex.) present there: 11.24 mm; width: 17.40 mm). At the site corresponding to the position of M1 on the left side, the surface of the alveolar process is smooth. No signs of inflammatory resorption, post-traumatic remodeling, or features indicating tooth loss during the individual’s lifetime are observed. The row of premolar alveoli is complete on both sides. Slight asymmetry in the length of the dental arch is observed (P1–P4: right side 53.31 mm; left 51.50 mm). The premolars (P4) are present on both sides and exhibit normal dimensions, falling within the range of population variation (P4 length: 19.57 mm on the right side and 19.07 mm on the left side).

### 3.8. Occlusion and Tooth Positioning

The development and arrangement of the preserved premolars and molars on the right side reveal fully formed roots, no signs of hypercementosis, and correct vertical orientation after establishing a horizontal line from the center of the distal zygomatic process of the zygomatic arch at the level of the zygomaticomaxillary suture to the *Prosthion* (Figure 6).

No displacement of adjacent teeth toward the gap left by the canine tooth was observed, as would be characteristic of post-traumatic loss resulting from collapse of the compact bone (*lamina dura*). This strongly supports the hypothesis of a missing primary tooth or its very early loss rather than late extraction.

#### 3.8.1. Incisor Region and Developmental Anomalies

The shapes of the left and right palatal fissures are not analogous. The right incisive bone shows a complete absence of alveolar structures corresponding to the incisors (I1–I2), with no visible alveoli or interdental septa. The bone surface is continuous and shows no morphological indicators of prior tooth presence or post-extraction remodeling. In contrast, the left incisive bone retains visible alveoli for teeth I2–I3 and evidence of the absence of tooth I1, as well as partial interdental architecture, despite moderate taphonomic alteration of the surface.

This marked asymmetry strongly suggests unilateral incisor agenesis on the right side. At the site of the I3 alveolus, a distinct depression with a rough-textured floor is visible (Figure 1). Compared to the smooth margins of the adjacent, empty alveoli, this site appears to be partially filled with spongy bone tissue or undergoing a remodeling process. This may suggest the loss of incisor I3 long before the animal’s death, which initiated a healing process and the slow overgrowth of the alveolar socket with new bone tissue. The visible depression is a trace of the remaining alveolar walls, which have not yet completely resorbed, for example, due to inactivity. The possibility of a prior inflammatory process affecting this site cannot be excluded, which affected the bone structure even before the tooth was lost.

The I3 incisor in canids is usually the largest of the incisors, and its alveolar socket is the deepest, which means that the process of its complete overgrowth takes the longest. The rough texture of the depression’s floor may indicate a past inflammatory process in the bone that occurred before the tooth fell out or was lost due to biting hard objects, which in a dog of this age (7–10 years) can occur as a result of weakened tooth attachment. A shallow floor may be evidence that the dog’s body attempted to repair the bone, and the fact that the depression is still visible indicates that the process of alveolar resorption has not yet been completed (or was hindered by a past inflammatory condition).

#### 3.8.2. Functional and Dietary Interpretation

This finding is noteworthy, as a dog with such defects would have had to cope with food intake despite the absence of a key incisor. In the urban environment of Wolin between 1100 and 1150, this may indicate human care or easy access to soft food remains. The degree of cuspal wear and the exposure of dark secondary dentin on the P4 premolar and M1 molar indicate a diet rich in abrasive components or frequent gnawing of very hard materials (for example, large mammal bones or antlers). Despite this advanced wear, the absence of open root canals suggests that the process was gradual, allowing for the deposition of secondary (defensive) dentin.

#### 3.8.3. Radiographic Assessment of Sutures

Based on the available projections, the median palatine suture (*sutura palatina mediana*) is partially open in its anterior portion (Figure 2), indicating that the individual was not of advanced age. Together with the maxillary and palatine sutures, which are not fully obliterated and retain relatively distinct margins, as well as the observed dental wear, this suggests a biological age of adultus.

#### 3.8.4. Macroscopic and Radiographic Findings of the Maxilla

Alveolar Defect and Taphonomic Interpretation

**Figure 7:**
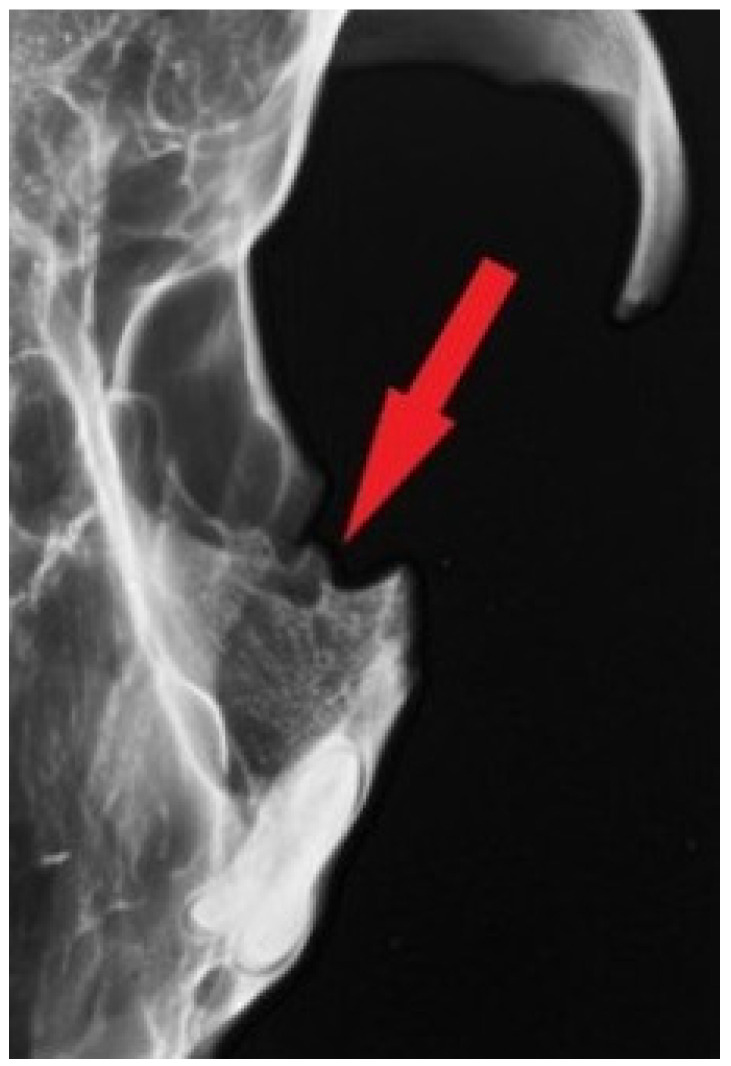
A section of the mandibular alveolar process containing the alveolus for M1.

The photograph shows a distinct defect in the distal portion of the alveolar arch (Figure 7).

The alveolar margins are rounded and do not exhibit sharp resorptive edges. In turn, the X-ray image reveals the absence of a structure corresponding to M^1^/M^2^, the absence of a root shadow, and partial pneumatization and remodeling of the maxillary bone at this site. Taking into account the taphonomic-developmental interpretation, there are no features of recent loss—the characteristic “open” alveolus. This suggests a process of long-term remodeling. This may result from the absence of the tooth bud or tooth loss at a young age, followed by complete remodeling.

#### 3.8.5. Canine Region: Macroscopic and Radiographic Correlation

Anatomical identification in the macroscopic photograph (Figure 1) on the left side, in the area indicated by the green arrow at the anatomical location of the maxillary canine, reveals the absence of a preserved tooth crown and an irregular bone surface without a clearly formed, open cylindrical alveolus. The observed trabecular structure, similar to cancellous bone, does not correspond to the wall of an active alveolus.

#### 3.8.6. Radiographic Characteristics of the Impacted Canine

**Figure 8:**
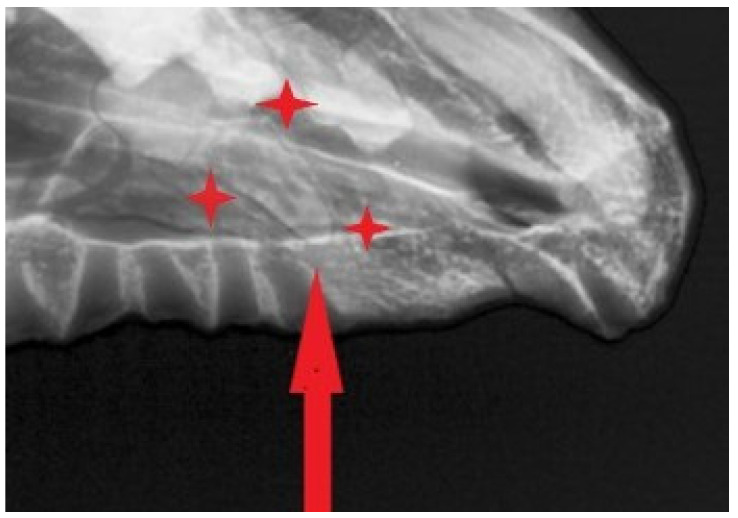
The arrow and stars indicate the outline of the impacted canine tooth within the maxilla.

The X-ray images in the dorsoventral (DV) projection of the skull (Figure 2), as well as the right and left lateral oblique projections (Figure 3 and Figure 4), revealed the presence of an unerupted, well-formed canine located within the maxilla. The crown and root of the tooth are clearly defined and show no signs of resorption, hypoplasia, or pathological structural changes (Figure 8).

The long axis of the tooth runs obliquely relative to the axis of the skull, and the tooth does not make contact with the oral cavity. At the site corresponding to the physiological position of the canine alveolus on the surface of the alveolar process, no bony opening is present, indicating alveolar hypoplasia rather than secondary tooth loss.

#### 3.8.7. Periapical Pathology of the Molar Region

The radiographic image reveals no signs of osteomyelitis, cysts, or remodeling suggestive of an acquired pathological process. The section of radiograph (Figure 7) shows alveolar bone defects at the root apices of the last molar on the right side. This indicates a chronic inflammatory process that was ongoing during the animal’s lifetime, which may have resulted from infection of the dental pulp, as the thin layer of dentin did not act as a barrier to microorganisms. Subsequently, the inflammatory process spread to the periapical tissues of the tooth roots, destroying the cortical bone.

#### 3.8.8. Asymmetry and Spatial Relationships

The observed asymmetry affects only the left side of the maxilla and includes: the presence of an impacted canine C, the absence of the M1 alveolus, and a shortened dental arch compared to the right side. The remaining bony structures, including the palate (greatest palatal breadth: measured across the outer border of the alveoli—max = 60.79 mm and last palatal breadth: measured behind the canines = 31.99 mm) and the viscero-cranial bones, show no significant deformities or secondary asymmetries.

### 3.9. Integrated Interpretation of Developmental Anomalies

Combined osteometric data, macroscopic observations, and radiological findings reveal the coexistence of two developmental anomalies: (1) a fully formed, impacted maxillary left canine without a corresponding alveolar process, and (2) agenesis (or early-stage developmental abnormalities) of the maxillary left first molar and right incisors, as evidenced by the complete absence of corresponding sockets. The absence of inflammatory markers, secondary infections, or advanced pathology—combined with the absence of post-traumatic or degenerative changes—suggests that these conditions were congenital. Furthermore, the available evidence does not indicate a major impairment of masticatory function.

## 4. Discussion

### 4.1. Diagnostic Assessment of Tooth and Alveolar Absence

The coexistence of a retained maxillary canine and the absence of developed alveoli for the first molar and incisors in the skull of the dog from Wolin analyzed here represents a rare case of dental developmental abnormalities requiring a definitive differential diagnosis.

In osteological material, the absence of a tooth or alveolus may result from several causes, including agenesis, arrest of tooth bud development, loss of the tooth during life, or taphonomic processes [46,55,56,84].

In cases of tooth loss following eruption, morphological traces of the alveolus or features of bone remodeling, such as surface irregularities, sclerotization, or foci of osteolysis, are usually preserved; radiological examinations may also reveal residual root structures or traces of their resorption. Healing and remodeling processes may lead to a partial reduction in alveolar socket depth; however, the boundary of the former dental crypt or irregularities in the bone surface usually remain recognizable. In the case under analysis, the absence of such structures, coupled with the continuity of the cortical layer and a homogeneous bone structure, is more consistent with the absence of primary tooth bud formation or its loss at a very early stage of development, prior to the formation of alveolar structures.

To systematize the diagnosis, a comparative matrix encompassing key morphological, taphonomic, and radiological features was applied (Table 4).

These criteria, based on paleopathological and clinical standards [13,46,84], allow for differentiation between post-eruptive tooth loss, agenesis, and early disruption of alveolar structure formation. In accordance with paleopathological recommendations, interpretation should be conducted within the context of the entire dental arch, taking into account symmetry, the presence of other anomalies, and spatial relationships between teeth. The application of these criteria reduces the risk of misinterpreting the absence of a tooth socket as tooth loss. Such an analysis was performed in the present case. On this basis, the observed features can be further interpreted in a broader clinical and comparative context.

### 4.2. Interpretation of Canine Impaction in a Comparative Context

In the analyzed case, the absence of the M1 alveolus, the presence of a smooth bone surface without features of reactive remodeling, and the absence of signs of inflammation argue against tooth loss during the animal’s lifetime. The absence of sharp bony edges and taphonomic fractures rules out post-mortem loss. The radiographic presence of a fully formed canine strongly argues against agenesis and may indicate tooth impaction. A comparison of the observed features with the diagnostic criteria listed in Table 4 allows for the diagnosis of tooth impaction of a congenital or very early developmental nature, although the absence of the M1 alveolus may reflect a very early ontogenetic disturbance prior to the full formation of the alveolar process.

Clinical and osteoarcheological literature indicates that maxillary canines are particularly prone to impaction due to their long development period and complex migration trajectory [2,85,86]. These teeth may remain completely unerupted while retaining normal crown and root morphology, which makes their identification difficult without the use of imaging studies [7,10]. Similar phenomena have been described in human material, where the absence of an alveolus is not always an unambiguous indicator of agenesis [35,87]. From a comparative perspective, these data support the interpretation of the observed changes as early developmental disorders. These observations warrant consideration of the underlying developmental mechanisms responsible for the observed anomalies.

### 4.3. Developmental Mechanisms and Biological Implications

From a developmental perspective, the observed combination of tooth agenesis and canine retention most likely reflects disturbances occurring in the early stages of odontogenesis. Tooth development in mammals is regulated by epithelial–mesenchymal interactions within the dental lamina, controlled by developmental genes such as MSX1, MSX2, and PAX9, as well as by signaling pathways involving BMP, FGF, SHH, and WNT [26,35,88]. Disruptions of these regulatory mechanisms during the early stages of embryogenesis can lead to the inhibition of the development of individual tooth buds, resulting in hypodontia or agenesis and preventing the formation of a recognizable alveolar socket. Teeth with a long and complex eruption trajectory, such as maxillary canines, are particularly susceptible to eruption disorders and may remain impacted despite the full formation of the crown and root [2,41,86]. Furthermore, according to developmental field theory, teeth belonging to the same morphogenetic region of the maxilla may be affected simultaneously, as their development is controlled by partially overlapping spatial and genetic mechanisms [89,90]. The combination of anomalies observed in the specimen from Wolin may therefore reflect a local disturbance of the maxillary odontogenic field rather than independent pathological events. In practice, this means that the developmental disturbance could have occurred at an early stage of dental lamina formation, before the bony architecture of the alveolar process was fully established. The most likely explanation is therefore an early odontogenic disorder affecting the maxillary dental field.

The absence of secondary inflammatory, resorptive, or degenerative changes within the jaw, combined with the animal’s advanced age (approximately 7–10 years), indicates that the observed anomalies did not significantly affect the functioning of the masticatory system or the animal’s survival. These data suggest that the described abnormalities have limited functional significance.

It should be emphasized, however, that the presented material constitutes a single case study. Based on a single documented individual, it is not possible to draw conclusions regarding the frequency of similar anomalies in the Wolin dog population or their significance in a broader breeding or social context. Interpretations extending beyond the biological level must be cautious and hypothetical.

The analyzed case indicates that the absence of a tooth socket in archeological material cannot be automatically equated with tooth loss. Each such case requires radiological examination for differential diagnosis. This case fits within the broader spectrum of developmental variability in dentition observed in mammalian populations and underscores the need for systematic documentation of similar anomalies in archaeozoological studies.

## 5. Conclusions

The skull 2493 AR955/7 from early-medieval Wolin documents the coexistence of an unerupted maxillary canine and the absence of the alveolar socket for the first molar on the same side, as well as the absence of the incisors. Macroscopic and radiographic evidence allows us to rule out post-mortem tooth loss and inflammatory destruction and most strongly supports a congenital or very early developmental origin of the observed changes.

The absence of secondary pathologies within the maxilla and the mature age of the individual indicate that the described abnormalities did not lead to significant impairment of masticatory function or reduced survival. This case highlights the importance of radiographic examinations in the analysis of archeological dentition and indicates that the absence of a tooth socket should not be automatically interpreted as tooth loss.

Due to the single-case nature of the material, the conclusions are limited in scope and cannot be extrapolated to the population level. Further studies involving larger comparative samples and the use of advanced imaging methods, such as computed tomography, are necessary for a more complete understanding of the etiology and prevalence of similar anomalies in historical dog populations.

## 6. Recommendations

The described case is rare, and there is a significant methodological gap due to a lack of standardization. The findings of Losey et al. [42] and Schernig-Mráz et al. [43] demonstrate the need for more detailed, imaging-based dental studies of archeological dogs. This work may serve as a reference case study for future research and comparisons, for example, within a comparative project encompassing Baltic collections and studies integrating animal health, archaeozoology, and human–animal welfare.

Radiography is a useful, non-invasive method for assessing dentition in excavated skeletal material, enabling the evaluation of root length, spatial arrangement within the maxilla, and the identification of pathological changes.

The primary projection in dental assessment should be the lateral view, in which the skull is positioned on its side and the X-ray beam is directed perpendicularly through its center. This projection enables the evaluation of root length and general tooth orientation within the maxilla.

The dorsoventral view, obtained with the skull positioned on the palate, allows assessment of dental arch symmetry. For detailed evaluation, particularly in cases of suspected agenesis, oblique projections are recommended to reduce the superimposition of bony structures.

For comprehensive and comparable radiological documentation, at least two orthogonal projections (lateral and dorsoventral) should be obtained, supplemented with oblique views as required for diagnostic purposes.

## Figures and Tables

**Table 1:** Metric characteristics of a dog skull from Wolin Island, dated to 1100–1150. Basic measurements follow Angela von den Driesch (1976) [80], with particular emphasis on the braincase. Measurement numbers 1–40 according to von den Driesch (1976) [80].

Osteometrics (mm)		x
1	Total length	182.00
2	Condylobasal length	171.00
3	Basal length	162.00
4	Basicranial axis	44.45
4a *	From the base of the foramen magnum to the suture pterygoid/palatine, where the palatine meets the presphenoid	52.59
4b *	From the basal part of the foramen magnum to the suture palatine/maxilla	101.67
5	Basifacial axis	115.75
6	Neurocranium length	101.00
7	Upper neurocranium length	88.66
11	Length of braincase (*Basion–Ethmoideum*)	81.62
22	Greatest diameter of auditory bulla	21.94
23	Greatest mastoid breadth	64.00
24	Breadth dorsal to the external auditory meatus	60.84
25	Greatest breadth of the occipital condyles	34.43
26	Greatest breadth of the bases of the paraoccipital processes	62.68
27	Greatest breadth of the foramen magnum	18.68
28	Height of the foramen magnum	14.18
29	Greatest breadth of the braincase	59.12
30	Zygomatic breadth	105.86
31	Least breadth of skull	38.25
32	Frontal breadth	47.49
33	Least breadth between orbits	33.80
37	Greatest inner height of the orbit	33.89
38	Skull height	53.28
39	Skull height without the sagittal crest	43.13
40	Height at the occipital triangle	36.52
42 * ♠	Neurocranium capacity	35.10
45 * ♥	Foramen magnum area	176.25
46 * ♦	Occipital triangle area	1144.54

Explanation: * Not included in Angela von den Driesch (1976) [80]; ♠ (A–N) × (Eu–Eu) × (CH); ♥ The area of the foramen magnum was determined using MultiScan Base 18.03 (license no. 172/12/12/34). The skull was positioned vertically (rostrum downward) under a digital camera (Sony Alpha 100, Sony, Tokyo, Japan) equipped with a Tamron SP Di AF 90 mm f/2.8 Macro 1:1 lens (Tamron, Saitama, Japan) and mounted on a calibrated stand. The plane of the foramen magnum was aligned perpendicular to the camera axis. The outline of the foramen magnum was approximated by an ellipse, and the area was calculated accordingly. ♦ The area of the occipital triangle was calculated as P = a × h/2, where *a* is the maximum width between the paracondylar processes (26) and *h* is the height from the Basion to the external occipital protuberance (40).

**Table 2:** Basic measurements of the dog viscerocranium. Measurement numbers 8–41 according to Angela von den Driesch (1976) [80].

No.	Osteometrics (mm)	x
8	Viscerocranium length	89.88
9	Facial length	105.16
10	Greatest length of the nasals	74.52
12	Snout length	81.95
13	Median palate length	91.22
13a	Palatal length	90.01
14	Length of the horizontal part of the palatine	32.67
14a	Length of the horizontal part of the palatine (corresponding to 13a)	31.69
15	Length of cheektooth row (d)	63.57
15a	Length of cheektooth row (s)	64.65
16	Length of molar row (d)	13.40
16a	Length of molar row (s)	-
17	Length of the premolar row (d)	53.31
17a	Length of the premolar row (s)	51.50
19	Length of carnassial alveolus (d)	19.54
19a	Length of carnassial alveolus (s)	18.95
19b	Length of P^4^ (d)	19.57
19c	Greatest breadth of P^4^ (d)	9.81
19d	Breadth of P^4^ (d)	7.62
19e	Length of P^4^ (s)	19.07
19f	Greatest breadth of P^4^ (s)	10.59
19g	Breadth of P^4^ (s)	8.28
20	Length of M^1^ (d)	11.24
20a	Breadth of M^1^ (d)	17.40
20b	Length of M^1^ (s)	
20c	Breadth of M^1^ (s)	-
21	Length of M^2^	-
21a	Breadth of M^2^	-
34	Greatest palatal breadth	60.79
35	Least palatal breadth	31.99
36	Breadth at the canine alveoli	31.85
41	Height of the canine	9.41
43 * ♠	Maxillofacial width	42.77
44 * ♦	Zygomatic length	94.96

Explanation: * Not included in Angela von den Driesch (1976) [80]. ♠ Facial width (maxillofacial width) was estimated as the distance between the lowest points of the zygomaticomaxillary sutures on both sides of the maxilla (*sutura zygomaticomaxillaris sinistra* and *dextra*). ♦ Zygomatic arch length was measured from the *Otion* to the most rostral point of the zygomaticomaxillary suture.

**Table 3:** Cranial indexes of dog skull found on Wolin Island, dated to the period 1100–1150.

Indexes		x
1	Zyg–Zyg ∗ 100/A–P	58.16
2	Eu–Eu ∗ 100/A–N	64.18
3	Zyg–Zyg ∗ 100/N–P	117.78
4	Eu–Eu ∗ 100/A–P	32.48
5	Eu–Eu ∗ 100/B–P	36.49
6	Eu–Eu ∗ 100/Condylobasal length	34.57
7	N–B ∗ 100/B–P	62.35
8	Pm–Pd ∗ 100/St–P	58.15
9	(A–N) ∗ (Eu–Eu) ∗ (CH)	35.10
10	B–S ∗ 100/P–S	38.40
11	Ect–Ect ∗ 100/A–P	26.09
12	N–A ∗ 100/N–P	102.49
13	Eu–Eu ∗ 100/F(So)–O	74.23
14	Palatal width ∗ 100/Greatest palatal length	67.53
15	Canine width/Palatal length	0.1151
16	Area *Foramen magnum*/Area of *occipital triangle*	15.40
17	Area of *occipital triangle*/Ot–Ot	17.88
18	B–P/Area of *occipital triangle*	14.15
19	P^1^–M^2^/A–P	29.14
20	Coefficient Wyrost–Kucharczyk = (1016 ∗ B–E) − 31.2	51.81
21 ♠	(*Maxillofacial* width)^2^/B–P	11.29
22	Forehead Width Index Ect–Ect/B–P	29.31
23	Exponential Forehead Width Index (Ect–Ect)^2^/B–P	13.92
24	Projected Viscerocranial Length (P–Ect) ∗ 100/B–P	65.64
25	Orbital Position Index (P–Ect ∗ 100/A–Ect)	116.13
26	Braincase Length Index (A–Ect) ∗ 100/B–P	56.52
27 ♥	Forehead Position Index P–So ∗ 100/So–O	126.69
28	Tooth Length Index P^1^–M^2^/B–P	43.09
29	Exponential Tooth Length Index (P^1^–M^2^)^2^/B–P	30.09
30 ♦	Index Zygomatic Arch Length/A–Zyg	1.07
31	Index Facial Skull Length N–Rh/N–P	0.83
32	Index1 Eu–Eu/N–Rh	0.79
33	Index 1a Eu–Eu/N–P	0.65
34	Index2 P–A/Zy–Zy	1.72
35	Index 2a P–B/Zy–Zy	1.53
36	Index 3 A–N/Zy–Zy	0.87
37	Index 4 A–N/Eu–Eu	1.56

Explanation: ♠ Facial width (maxillofacial width) was estimated by measuring the distance between the lowest points of the zygomaticomaxillary sutures on both sides of the maxilla (*sutura zygomaticomaxillaris sinistra* and *dextra*). ♥ Forehead position index = (P–So) × 100/(So–O), where So (*Sagectorbion*) is the point of intersection between the sagittal suture and the line connecting the most lateral points of the zygomatic supraorbital processes of the frontal bones. ♦ Zygomatic arch length was measured from the *Otion* to the most rostral point of the zygomaticomaxillary suture. The asterisk (∗) in the table is used to indicate multiplication.

**Table 4:** Differential diagnosis of missing or non-erupted teeth in osteological material with reference to the Wolin specimen.

Feature	Agenesis	Early Developmental Arrest	Early-Life Tooth Loss	Post-Mortem Loss
Presence of socket	absent	absent or poorly formed	initially present	present
Bone remodeling	smooth surface	smooth surface	reactive remodeling possible	sharp edges
Inflammatory signs	absent	absent	may be present	absent
Radiographic root shadow	absent	absent	absent	absent
Adjacent tooth displacement	absent	absent	possible	absent
Taphonomic cracks	no	no	no	possible

## Data Availability

Raw DICOM images and osteometric measurement data are available from the corresponding author upon reasonable request.
